# Posttraumatic stress disorder symptom severity and control beliefs as the predictors of academic burnout amongst adolescents following the Wenchuan Earthquake

**DOI:** 10.1080/20008198.2017.1412227

**Published:** 2017-12-18

**Authors:** Xiao Zhou, Rui Zhen, Xinchun Wu

**Affiliations:** ^a^ Beijing Key Laboratory of Applied Experimental Psychology, Faculty of Psychology, Beijing Normal University, Beijing, China; ^b^ I-Core Research Center for Mass Trauma, Bob Shapell School of Social Work, Tel Aviv University, Tel Aviv, Israel

**Keywords:** Adolescents, PTSD, control beliefs, academic burnout, adolescentes, TEPT, creencias de control, desgaste académico, 青少年, PTSD, 控制信念, 学业倦怠, • The combined role of PTSD and control beliefs in academic burnout was examined. • PTSD symptom severity was associated positively with academic burnout.• Primary and secondary control beliefs predicted negatively academic burnout.• Primary control beliefs buffered the positive effect of PTSD on academic burnout.

## Abstract

**Background**: Previous studies indicate that posttraumatic stress disorder (PTSD) and control beliefs can affect burnout and their unique role in this situation has been examined, but fewer studies have examined their combined role in adolescent’s academic burnout following traumatic events.

**Objective**: This study examined the combined effect of PTSD symptom severity and control beliefs on academic burnout among adolescents, and assessed the moderating role of primary and secondary control beliefs in the relation between PTSD symptom severity and academic burnout.

**Methods**: Seven hundred and forty-five adolescents were investigated using self-report questionnaires, and a series of regression equations examined the impact of PTSD severity and control beliefs on academic burnout.

**Results**: PTSD symptom severity is associated positively with academic burnout, while primary and secondary control beliefs have a negative relation with academic burnout. In addition, primary control beliefs buffer the positive effects of PTSD symptom severity on academic burnout. We found that the positive relation between PTSD symptom severity and academic burnout in the low primary control beliefs group is more intense than that found in the high primary control beliefs group.

**Conclusions**: PTSD symptom severity is a risk factor, whereas primary and secondary control beliefs are protective factors in academic burnout. In addition, PTSD symptom severity and primary control beliefs have a combined effect on academic burnout in adolescents following natural disasters.

## Abstract (es)

**Planteamiento:** Estudios previos indican que el trastorno por estrés postraumático (TEPT) y las creencias de control pueden afectar al agotamiento y se ha examinado su singular papel en esta situación, pero menos estudios han examinado su papel combinado en el desgaste académico de los adolescentes después de eventos traumáticos.

**Objetivo:** Este estudio examinó el efecto combinado de la gravedad de los síntomas de TEPT y las creencias de control sobre el desgaste académico entre adolescentes y evaluó la función moderadora de las creencias de control primarias y secundarias en la relación entre la gravedad de los síntomas de TEPT y el desgaste académico.

**Métodos:** Se estudiaron 740 adolescentes utilizando cuestionarios de autoinforme, y una serie de ecuaciones de regresión examinaron el impacto de la gravedad del TEPT y las creencias de control sobre el desgaste académico.

**Resultados:** La gravedad de los síntomas del TEPT se asocia positivamente con el desgaste académico, mientras que las creencias de control primarias y secundarias tienen una relación negativa con el desgaste académico. Además, las creencias de control primarias amortiguan los efectos positivos de la gravedad de los síntomas del TEPT en el agotamiento académico. Encontramos que la relación positiva entre la gravedad de los síntomas de TEPT y el desgaste académico en el grupo con pocas creencias de control primarias es más intensa que la encontrada en el grupo de elevadas creencias de control primarias.

**Conclusiones:** La gravedad de los síntomas de TEPT es un factor de riesgo, mientras que las creencias de control primarias y secundarias son factores de protección en el desgaste académico. Además, la gravedad de los síntomas del TEPT y las creencias de control primarias tienen un efecto combinado sobre el desgaste académico en adolescentes después de un desastre natural.

## Abstract (zh)

**背景：**前人研究提示创伤后应激障碍（PTSD）和控制信念会影响倦怠。虽然二者各自的作用已经被考察过，但是很少研究关注它们对经历过创伤事件的青少年的学业倦怠的共同作用。

**目标：**本研究旨在考察PTSD症状严重程度和控制信念对青少年的学业倦怠的共同作用。同时考察首要和次要控制信念在PTSD 症状严重程度和学业倦怠之间的调节作用。

**方法：**745名青少年填写了自评问卷，并使用一系列回归方程对PTSD 症状严重程度和控制信念对学业倦怠的影响进行分析。

**结果：**PTSD症状严重度和学业倦怠正相关，而首要和次要的控制信念和学业倦怠之间有负相关。另外，主要控制信念缓冲了PTSD 症状严重程度对学业倦怠的正向影响。我们发现在低主要控制信念组和高主要控制信念组相比，PTSD 症状严重程度和学业倦怠的正相关性更加显著。

**结论：**对学业倦怠来说，PTSD 症状严重程度是一个风险因素，而主要控制信念和次要控制信念是保护性因素。另外，在经历自然灾害的青少年中，PTSD 症状严重程度和主要控制信念对学业倦怠有一个相加的效果。

## 1. Introduction

Trauma-related studies suggest that posttraumatic stress disorder (PTSD) is one of the common negative psychological outcomes following traumatic events (Breslau, Troost, Bohnert, & Luo, 2013; Carmassi et al., 2013). Particularly, the prevalence rate of PTSD among adolescents is higher than adults (Hafstad, Dyb, Jensen, Steinberg, & Pynoos, 2014; Wang, Long, Li, & Armour, 2011), as adolescence is a crucial juncture from both physical and psychological perspectives (Salmela-Aro, Savolainen, & Holopainen, 2009).

In addition to PTSD, in terms of the education environment, traumatic events, especially those natural disasters that cover a wide range of areas, can produce uncertain and insecure school settings. These can lead to a mismatch between adolescent needs and available resources, and in turn make it possible for adolescents to experience academic burnout (Ying, Wang, Lin, & Chen, 2016). Here, academic burnout refers to a syndrome marked by emotional exhaustion, cynicism, and academic inefficacy resulting from the continued failure to successfully cope with academic stress (Schaufeli, Martinez, Pinto, Salanova, & Bakker, 2002). Studies have recently suggested that students who have experienced major life events are inclined to have more academic burnout (Dyrbye et al., 2006; Ying et al., 2016), and thus academic burnout can also be found in students who have had traumatic experiences (Xu et al., 2017). That is, adolescents not only show PTSD, but they also report academic burnout following traumatic events, and some researchers have suggested that PTSD and burnout may be coexistent in people in higher stressful environments (Mealer, Burnham, Goode, Rothbaum, & Moss, 2009). Thus, a question arises: is there a relation between these two negative psychological outcomes following trauma? To answer this question, our study examined the relation between PTSD symptom severity and academic burnout among adolescents following traumatic events.

To our knowledge, no studies to date have examined the relation between PTSD symptom severity and academic burnout among adolescents. Nevertheless, many studies have focused on the association between PTSD symptom severity and job burnout in adults (Baird & Jenkins, 2003; Mealer et al., 2009; Mitani, Fujita, Nakata, & Shirakawa, 2006); generally finding a positive association between these (Boudoukha, Altintas, Rusinek, Fantini-Hauwel, & Hautekeete, 2013; Rojas-Flores et al., 2015). One potential explanation for this pattern is that PTSD itself is a kind of emotional distress that is characterized by increased hyper-arousal states; people with more serious PTSD symptoms may be experiencing higher emotional tension and vigilance (Sullivan & Elbogen, 2014), and thus this type of person may be more likely to feel emotional exhaustion. Due to this, people with PTSD have reported more burnout compared to those without PTSD (Boudoukha et al., 2013; Rojas-Flores et al., 2015).

Another potential explanation may be attributed to the demand–resource model (Demerouti, Bakker, Nachreiner, & Schaufeli, 2001; Schaufeli & Bakker, 2004). This model assumes that every occupation may have two categorical factors (demands and resources) related to burnout: when lack of resources complicates the meeting of demands; then burnout may be present (Demerouti et al., 2001). Also, individuals with more serious PTSD symptoms may have fewer resources to meet their demands at work, and thus this situation predisposes them to experience burnout (Rojas-Flores et al., 2015). Because students’ core activities may be considered as a kind of ‘work’ (Shih, 2012; Zhang, Klassen, & Wang, 2013), we may speculate that PTSD symptom severity will be associated positively with academic burnout among adolescents.

The demand–resource model also suggests that the availability of resources leads to commitment and engagement (Xanthopoulou, Bakker, Demerouti, & Schaufeli, 2007), thus studies have recently suggested that resources may play a part in an extrinsic or intrinsic motivational process, and foster individuals’ ability to meet their goals (Hackman & Oldham, 1980; Schaufeli & Taris, 2014), protecting them from burnout. Thus, the demand–resource model is more inclined to emphasize the role of personal resources given that it is functional in accomplishing work goals (Schaufeli & Taris, 2014), but also claims that individuals’ sense of their ability to control their environment has a more important role in burnout (Xanthopoulou et al., 2007). Thus, it is more likely that control beliefs are another factor related to burnout (Glass & McKnight, 1996; McKnight & Glass, 1995).

Control beliefs refer to the belief system under which individuals perceive that they have some control over life events (Xin, Zhao, & Guo, 2008). Control beliefs cover two categories: primary and secondary control beliefs. The former, consisting of direct and indirect control beliefs, means that people develop a sense of personal control by direct or indirect manipulating of external, objective conditions or environment to fit their needs and wants (Weisz, 1990). In contrast, emotional and cognitive control beliefs constitute secondary control beliefs, which refer to people changing their internal conditions, including cognition and emotions, to accommodate existing reality (Chang, Chua, & Toh, 1997).

Both primary and secondary control beliefs have an adaptive function (Heckhausen, Wrosch, & Schulz, 2010). Specifically, primary control beliefs drive people to invest time and effort in goal attainment or overcoming problems (Heckhausen et al., 2010), and people who succeed in achieving goals or solving problems will perceive a better sense in coping with plight (Band, 1990), and healthier outcomes (Barlow, Liu, & Wrosch, 2015; Hall, Chipperfield, Heckhausen, & Perry, 2010). In turn, they experience less burnout (Glass & McKnight, 1996). However, it is noteworthy that primary control beliefs may be maladaptive in the context of failure and stress, because goals are unattainable and resources are depleted. In this situation, people may resort to secondary control beliefs, which can contribute to adaptive outcomes by reducing emotional distress or facilitating disengagement from unattainable goals (Heckhausen et al., 2010). It has also been suggested that secondary control beliefs are related to less depression, less loneliness (Barlow et al., 2015; Compas et al., 2006), and more positive moods (Ying et al., 2014), which protects people from burnout. Thus, it is more likely that control beliefs can relieve burnout.

While previous studies have suggested that primary and secondary control beliefs may reduce burnout, the demand–resource model also suggests that personal resources may play a buffering role in the relation between stress and burnout (Bakker, Demerouti, & Euwema, 2005). Because of these personal characteristics, people may alter the perceptions and cognitions evoked by stressors, buffer the responses following appraisal processes, and reduce the consequences of responses that damage health (Kahn & Byosiere, 1992), thus protecting themselves from burnout. Because control beliefs are an important personal characteristic or resource (Hobfoll, Johnson, Ennis, & Jackson, 2003), and PTSD itself indicates serious stress, we proposed in our study that control beliefs may buffer the relation between PTSD symptom severity and burnout. For example, compared to individuals with low primary control beliefs, individuals with high primary control beliefs may try to take an active approach toward change or the management of a stressful event (Ying et al., 2014), which in turn helps traumatized individuals change their focus from the negative aspects of trauma to positive ones (Nes & Segerstrom, 2006). Individuals with PTSD may also engage in positive behaviours, and thus the exacerbating role of PTSD in other negative consequences (e.g. burnout) may be buffered. Compared to individuals with low secondary control beliefs, individuals with high secondary control beliefs may change their cognition and emotion to accommodate existing reality (Chang et al., 1997). This may help traumatized individuals to make sense of traumatic events, and to rediscover the significance of the world following their trauma. Finally, this may also buffer the effect of PTSD on other negative outcomes (e.g. burnout). However, these propositions are explained theoretically only; no empirical studies to date have examined them. Carrying out studies involving the moderating role of control beliefs in the relation between PTSD and academic burnout will be beneficial to the intervention of academic burnout in students following traumatic events.

However, previous studies have focused on the relation between PTSD symptom severity and job burnout (Boudoukha et al., 2013; Rojas-Flores et al., 2015), and have overlooked the relation of PTSD to academic burnout in adolescents. Additionally, although the unique role of PTSD symptom severity and control beliefs in burnout has been assessed (Baird & Jenkins, 2003; Glass & McKnight, 1996), no studies to date have examined their combined role in burnout, particularly in academic burnout among adolescents. Furthermore, the demand–resource model is considered to apply to various occupational settings (Bakker et al., 2005), for example, in academic settings, students’ core activities may be considered as a kind of ‘work’ (Shih, 2012; Zhang et al., 2013), and they also encounter many different study demands and resource situations, which may later be manifested in their level of burnout at school (Salmela‐Aro & Upadyaya, 2014). However, few studies have examined the application of this model in studies of student behaviour (Salmela‐Aro & Upadyaya, 2014).

To fill these gaps, the aim of this study was to examine the role of PTSD symptom severity and control beliefs in academic burnout, and to assess the moderating role of control beliefs on the relation between PTSD symptom severity and academic burnout. Three hypotheses need to be addressed: (1) PTSD symptom severity is associated positively with academic burnout; (2) primary and secondary control beliefs is associated negatively with academic burnout; and (3) primary and secondary control beliefs may play a buffering role in the relation between PTSD symptom severity and academic burnout.

## 2. Method

### 2.1. Participants

The original sample consisted of 745 adolescents, but nine participants were excluded from further analysis because their proportion of missing data reached about 50%. The mean age of the 736 participants were 15.02 (*SD* = 1.64) years, ranging from 12.0 to 19.0 years; 406 (55.2%) were female, 326 (44.5%) were male, and four (0.5%) participants did not report their gender. All the participants experienced the Wenchuan earthquake, 21.7% of the participants were trapped, and 9.5% were injured in the earthquake.

### 2.2. Procedures

We conducted this study one year after this earthquake occurred, and Wenchuan and Mao County in Sichuan province were our focus because they were most severely affected by this earthquake. We firstly informed the local education authorities of the aims and methods of investigation in this study, and that we could provide psychological services if, and when, they were required. With the help of the local education authorities, we obtained the approval of several middle schools in the two counties. Then we randomly selected several classes to investigate. All students in selected classes attended school on the assessment date.

This project was approved by the Research Ethics Committee of Beijing Normal University and the local education authorities (the County Departments of Education), as well as the participating school principals. Written informed consent was obtained from school principals and each student. The purpose of the study and the autonomy of subjects were highlighted before the survey, and the right of each subject to withdraw from the survey at any time was explained. The assessment was conducted under the supervision of trained individuals with Master’s degree in psychology. The researchers administered the questionnaire packets in a classroom setting without the presence of teachers. After the questionnaire packets were completed, participants were told that school psychologists or teachers were available to provide any psychological/counselling services that they might need.

### 2.3. Measures

#### 2.3.1. Severity of trauma

The severity of adolescent survivors’ traumatic experiences was assessed by the trauma exposure questionnaire developed by Wu, Zhang, Lin, and Zang (2013). All 18 items of this questionnaire ask participants to indicate whether they had directly seen or indirectly heard about the death, injury or entrapment of parents, friends, teachers or others. Each of the items is rated on a 3-point scale, where 3 represents ‘saw by myself’, 2 represents ‘heard about through others’, and 1 represents ‘did not experience the situation above.’ In this study, the internal reliability of the questionnaire was good (α = 0.86).

#### 2.3.2. PTSD

The posttraumatic stress symptom level was assessed by the Chinese version (Zang, Zhang, & Wu, 2009) of the Child PTSD Symptom Scale (Foa, Johnson, Feeny, & Treadwell, 2001). This is a 17-item self-report scale designed to allow measurement of the occurrence and frequency of PTSD symptoms, in relation to the most distressing event. In the current study, all the items were translated into Chinese, and children rated the frequency of symptoms during the past 2 weeks on a 3-Point-Likert scale of 0 (*not at all*) to 3 (*almost always*). Subscale scores ranged from 0 to 15 for intrusion, 0 to 21 for avoidance, and 0 to 15 for hyperarousal. An overall score was generated by adding the scores of the three symptoms. In the current sample, the scale demonstrated a good internal consistency (α = 0.89).

#### 2.3.3. Control beliefs

Control beliefs were assessed using the Chinese version (Xin et al., 2008) of the PSCS (Chang et al., 1997). This scale has two subscales, namely primary and secondary control beliefs subscales, and each consists of two dimensions. The former, including direct and indirect control beliefs, measures the extent individuals attempt to exert direct or indirect control over a situation (e.g. ‘I find out the cause to deal directly with the problem’). The latter, including emotional and cognitive control beliefs, measures individuals’ attempts to change their affect and cognition to cope with stressors (e.g. ‘I accept that it has happened’). Each item is scored on a 5-point scale ranging from 0 (*not at all*) to 4 (*nearly all of the time*). In this study, the Cronbach alpha coefficient for the total scale was 0.94, and for primary and secondary control beliefs was 0.92 and 0.89, respectively.

#### 2.3.4. Academic burnout

Academic burnout was measured using the academic burnout inventory (ABI) developed by Hu and Dai (2007) on the basis of the theory of job burnout (Maslach, Schaufeli, & Leiter, 2001). The ABI consists of 21 items covering the three-dimension construct (i.e. emotional exhaustion, cynicism, and academic inefficiency). Each item is rated on a 5-point Likert scale ranging from 0 (*never*) to 4 (*always*). High total scores reflect high academic burnout levels. The ABI was found to have good internal consistency, and construct, convergent, and discriminant validity (Hu & Dai, 2007). In the current study, the Cronbach alpha coefficient for the total scale was 0.84.

### 2.4. Statistical analysis

The analyses were conducted using SPSS 17.0 software. First, we analysed the missing data in the questionnaires and found that this phenomenon was less than 8.4% across all variables. To assess the pattern of missing data, we used Little’s Missing Completely at Random (MCAR) test. The result revealed that the data were missing at random [χ^2^ (79) = 77.334, *p* = .532]. We therefore adopted linear imputation to handle missing data in the later regression analyses. Next, descriptive statistics were used to calculate the mean levels of the main measures, and Pearson’s correlations were used to assess the relations between age, gender, severity of trauma, PTSD symptom severity, primary and secondary control beliefs, and academic burnout. Then, to examine the moderating role of the two types of control beliefs on the relation between PTSD symptom severity and academic burnout, we conducted a series of hierarchical regression analyses by using the same procedures as Wen, Hau, and Chang (2005). Here, the predictive role of the independent variable and moderator should be examined in the first hierarchical regression analysis, and then the role of the interaction items between the independent variables and moderators should be assessed in a second set of hierarchical regression analyses (Wen et al., 2005).

In these analyses, the dependent variable was academic burnout, the independent variable was PTSD symptom severity, and the moderators were the two types of control beliefs (e.g. primary and secondary control beliefs). Moreover, independent and moderating variables were centred on their respective means to reduce multicollinearity between main effects and interactional terms, and to increase the interpretability of the weights for the interaction terms (Cohen, Cohen, West, & Aiken, 2013). In addition, gender, age, and severity of trauma were considered as compounding variables and controlled in each analysis, due to the relations between gender, age, and trauma severity and burnout (Galek, Flannelly, Greene, & Kudler, 2011; Lee, Puig, Lea, & Lee, 2013; Salmela-Aro & Tynkkynen, 2012). Specifically, we inserted compounding variables (e.g. age, gender, and severity of trauma) into the first hierarchical regression, then the PTSD symptom severity and primary control beliefs were included in the second hierarchical regression, and the interaction items between PTSD symptom severity and primary control beliefs included in a third hierarchical regression. Next, the same analytic procedures were used to examine the moderating role of secondary control beliefs.

Once we had determined that the interaction item between PTSD symptom severity and control beliefs has significant effects on academic burnout, we saw that control beliefs may play a potential moderating role in the relation between PTSD symptom severity and academic burnout. To further examine the significance of this potential moderating effect of control beliefs in the relation between PTSD symptom severity and academic burnout, we divided adolescents into high level (1 SD above the mean score of control beliefs) and low level belief groups (1 SD below the mean score of control beliefs). Soper’s (2009) simple slope analysis was adopted to further examine the significance of the moderating effect of control beliefs.

## 3. Results

### 3.1. Descriptive statistics and correlations among measures

Descriptive statistics and correlations among different measures are provided. As shown in Table 1, the mean levels of PTSD (*M* = 15.97, *SD* = 8.61), primary control beliefs (*M* = 26.06, *SD* = 10.95), secondary control beliefs (*M* = 29.51, *SD* = 10.51), and academic burnout (*M* = 32.48, *SD* = 13.34) were first assessed. Next, to examine the correlation between the main measures, and to decide whether age, gender, and trauma severity should be controlled, Pearson’s correlation was used. From Table 1, we see that gender is significantly associated with PTSD symptom severity; age is significantly associated with all variables; severity of trauma is significantly related to PTSD symptom severity and secondary control beliefs; PTSD symptom severity is related significantly to primary control beliefs and academic burnout; and primary control beliefs rather than secondary control beliefs are associated with academic burnout. These results indicate that gender, age, and trauma severity should be controlled in the subsequent analysis involving the moderating role of control beliefs in the relation between PTSD symptom severity and academic burnout.

**Table 1.** Means, standard deviations, and correlations of main measures.

	*M* (*SD*)	1	2	3	4	5	6
1. Gender	–	1.00					
2. Age	15.02 (1.64)	−0.02	1.00				
3. Severity of T trauma	24.49 (5.77)	0.04	0.09*	1.00			
4. PTSD symptom severity	15.97 (8.61)	0.22***	0.12**	0.26***	1.00		
5. Primary control beliefs	26.06 (10.95)	0.02	0.19***	0.05	0.10*	1.00	
6. Secondary control beliefs	29.51 (10.51)	0.06	0.29***	0.13**	0.07	0.75***	1.00
7. Academic burnout	32.48 (13.34)	0.01	0.23***	0.05	0.51***	−0.14***	−0.04

***p < .001; **p < .01; *p < .05.

### 3.2. Examination of the roles of PTSD symptom severity and control beliefs in academic burnout

Table 2 gives the results of the hierarchical regression analyses. The results show that, after controlling for gender, age, and trauma severity, PTSD symptom severity has a positive association with academic burnout, and primary and secondary control beliefs are associated negatively with academic burnout. The interaction between PTSD symptom severity and primary control beliefs has a negative association with academic burnout, but the interaction between PTSD symptom severity and secondary control beliefs has a non-significant role in academic burnout. These findings indicate that PTSD symptom severity is an important risk predictor of academic burnout, and control beliefs are protective factors in academic burnout. More importantly, primary rather than secondary control beliefs may play a moderating role in the relation between PTSD symptom severity and academic burnout.

**Table 2.** Regression analysis results: the effects of PTSD symptom severity and control beliefs on academic burnout.

Variables	B	*SE* B	*t*
Gender	0.62	0.02	0.62
Age	1.69	0.21	5.64***
Severity of trauma	0.06	0.03	0.68
PTSD	0.81	0.51	15.08***
Primary control	−0.26	−0.22	−6.78***
PTSD × Primary control	−0.01	−0.07	−2.06*
Gender	0.81	0.03	0.83
Age	1.78	0.23	6.02***
Severity of trauma	0.05	0.02	0.56
PTSD	0.77	0.50	14.86***
Secondary control	−0.16	−0.13	−4.10***
PTSD × Secondary control	0.00	−0.03	−0.93

***p < .001; *p < .05. PTSD = PTSD symptom severity, Primary control = primary control beliefs, Secondary control = secondary control beliefs.

### 3.3. Examination of the moderating role of primary control beliefs

**Figure 1.**
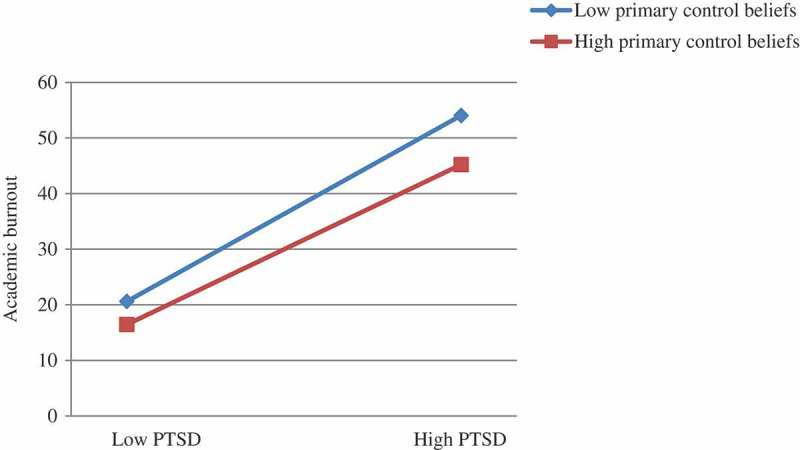
Interaction between PTSD and primary control beliefs on academic burnout. Note: PTSD = PTSD symptom severity

To further examine the significance of the interaction between PTSD symptom severity and primary control beliefs, we divided the adolescents into those having high primary control belief (1 SD above the mean score for primary control beliefs) and those having low primary control belief groups (1 SD below the mean score for primary control beliefs). Then, we used Soper’s (2009) simple slope analysis to examine the significance of the moderating effect of primary control beliefs. Figure 1 gives the results of this simple slope analysis; here we found that the interaction items between PTSD symptom severity and primary control beliefs negatively and significantly predict academic burnout [B = −0.008, *t* = −2.12, *p* < .05]. In the low primary control beliefs group, the relation between PTSD symptom severity and academic burnout (*SE* B = 0.66, *t *= 8.19, *p *< .001) was more intense than that found in the high primary control beliefs group (*SE* B = 0.56, *t *= 6.65, *p *< .001). The results indicate that primary control beliefs may buffer the positive association of PTSD symptom severity with academic burnout.

## 4. Discussion

To our knowledge, this is the first study to examine the effect of PTSD symptom severity and control beliefs on academic burnout, not only of their unique roles, but also of their combined effects. After controlling for gender, age, and trauma severity, the findings indicate that PTSD symptom severity is associated positively with academic burnout, but primary and secondary control beliefs are all negatively related with academic burnout. More importantly, our findings further suggest that primary control beliefs moderate the association between PTSD symptom severity and academic burnout.

Specifically, and consistent with previous studies on adult job burnout (Baird & Jenkins, 2003; Mealer et al., 2009; Mitani et al., 2006) and our first hypothesis, this study also found that PTSD symptom severity was an important risk factor for adolescents’ academic burnout in the posttraumatic event context. A possible explanation is that, after trauma, people need to spend much individual resources (i.e. cognitive or emotional resources) to relieve the distress from PTSD (Solomon & Dekel, 2007; Zhou, Wu, & Chen, 2015), and this will lead to resource depletion when having to cope with academic problems. The mismatch between individual resources and demands may finally lead to burnout (Rojas-Flores et al., 2015). One characteristic of PTSD, a hyperarousal state, can affect students with severe emotional tension (Sullivan & Elbogen, 2014) and result in emotional exhaustion. In general, PTSD can give adolescents more cognitive, emotional, and behavioural problems (Husain, Allwood, & Bell, 2008; Vasterling et al., 2002), and all of these factors will increase the probability of adolescent academic burnout.

Additionally, this study extended previous studies and elucidated the association between control beliefs and burnout by examining the role of primary and secondary control beliefs. The findings indicate that, after controlled gender, age, and severity of trauma, primary and secondary control beliefs are negatively associated with academic burnout, which supports the demand–resource model (Demerouti et al., 2001; Schaufeli & Bakker, 2004), and suggests that personal resources are the protective factors in academic burnout. To be specific, primary control beliefs can help students to seek solutions for coping with stressors (Heckhausen et al., 2010), particularly problem-focused solutions (Osowiecki & Compas, 1998), and in turn can improve their self-efficacy and positive psychological experiences when tackling such problems (Rahmati, 2015), and thus they are less likely to experience burnout in learning.

Another possible explanation was proposed to elucidate the negative relation between secondary control beliefs and academic burnout: secondary control beliefs may help students to accommodate the existing models in the cognitive world (Joseph, Linley, & Harris, 2005) to accept reality (McCracken, 1998). The acceptance of the world following traumatic events indicates changes in views on self, others, and the world (Janoff-Bulman, 2004); this will relieve negative psychological reactions (Åkerblom, Perrin, Fischer, & McCracken, 2015), and enhance posttraumatic event adjustment (Hayes, Luoma, Bond, Masuda, & Lillis, 2006). This in turn will relieve academic burnout. Taking these findings together, it is suggested that both primary and secondary control beliefs have an adaptive function for adolescents’ studies in the posttraumatic event context.

In addition, we found that primary rather than secondary control beliefs play a buffering role in the relation between PTSD symptom severity and academic burnout. Specifically, the positive relation between PTSD symptom severity and academic burnout in the low level group of primary control beliefs was closer than that in the high level group of primary control beliefs. Here, primary control beliefs inspire students to find directly or indirectly the cause of problems, and develop effective strategies to cope with problems by themselves (Xin et al., 2008). Thus, when they have a high level of primary control beliefs, students can cope with problems and experience higher self-efficacy (Rahmati, 2015), and this will protect them from developing academic burnout through the negative outcomes of PTSD. Nevertheless, this study did not find there to be moderating effects from secondary control beliefs in the relation between PTSD symptom severity and academic burnout. Here, Heckhausen et al. (2010) suggested that when certain goals become unattainable, secondary control strategies might play a buffering or protective role. In this study, however, adolescents are seen to attain their academic mission and reduce academic burnout by using primary control beliefs. Therefore, secondary control beliefs do not play a significant buffering role in the relation between PTSD symptom severity and academic burnout.

Several design and measurement limitations of this study must be acknowledged. First, all variables were measured by self-reported scales; future studies should consider gathering data by multiple methods. Second, the study was conducted with a sample of adolescent survivors after the Wenchuan earthquake in China, therefore its external validity may be limited, and its generalization to people with other traumatic experiences must be made with caution. In addition, PTSD symptom severity, control beliefs, and academic burnout may present differently due to different trauma types. This study only focused on these variables in adolescents following an earthquake, and overlooked the role of other traumatic events. Therefore, future studies would benefit from examining and comparing further the relation between PTSD symptom severity, control beliefs, and academic burnout in other forms of trauma. In addition, the trauma exposure questionnaire in our study did not completely assess the severity of trauma, and thus future studies should adapt these questionnaires with more items involving exposure to trauma when examining the severity of adolescent traumas.

Notwithstanding these limitations, the current study is of importance because, to the best of our knowledge, it is the first to examine the influences on adolescent academic burnout after an earthquake from the perspective of PTSD symptom severity and control beliefs. The findings indicate that PTSD symptom severity is the risk factor, while control beliefs are the protective factor adolescent academic burnout. Additionally, the results show that primary control beliefs can buffer the effect of PTSD symptom severity on academic burnout. From both educational practice and clinical perspectives, clinical efforts should therefore focus on decreasing PTSD symptom severity and improving adolescents’ control beliefs in school learning. Overall, interventions that concentrate on PTSD symptom severity, student control over outcomes, and the level of their subsequent academic burnout should be integrated into the existing systems of school mental health support.
